# Supplementary material for: Academic business research: Impact on academics versus impact on practice
Source: PLoS One. 2023 Dec 6;18(12):e0289034. doi: 10.1371/journal.pone.0289034 (PMC10699644; doi:10.1371/journal.pone.0289034)
Supplement: Attachment — Submitted filename: Response to Reviewers.docx [file pone.0289034.s001.docx]

Dear Academic Editor, Reviewer #1, and Reviewer #2

Thank you for your feedback and granting us an opportunity to revise the manuscript. We would like to believe that we have addressed all your concerns. We reproduce your comments in italics and write our responses in regular font.

**Authors’ Responses to Academic Editor’s Comments**

*Thank you for submitting your manuscript to PLOS ONE. After careful consideration, we feel that it has merit but does not fully meet PLOS ONE’s publication criteria as it currently stands. Therefore, we invite you to submit a revised version of the manuscript that addresses the points raised during the review process.*

Thank you for giving us the opportunity.

**Authors’ Responses to Reviewer #1’s Comments**

*You must be highly commended for a very good research topic. Actually no one , in my opinion, has researched the practical side of journals. Many scientists write articles for reputable journals and nobody wonders whether the models, methods etc. developed are applied in practice. Does the IF journal have an impact on the practical usefulness of the research results presented in journals?*

*I have done a lot of reviewing already, but this is the first time I am reviewing research results about the practical impact of journals. Soper, my congratulations to the authors for an interesting research topic. However, I have a few comments:*

1. *The abstract and keywords need to be expanded. In the abstract, the authors should add the* ***purpose*** *of the research, briefly* ***describe*** *the methodology and add the results, please do not limit yourself to only one top journal: American Economic Review, which, as the authors state, " leads all 56". It should be pointed out that there were 12 business disciplines (those in Figure 2) and more about the results.*

Thank you for suggesting these edits. The revised Abstract has all these edits.

*The following should be added or changed to the keywords: journal impact on practices, journal analysis, business disciplines*

We have added these three key terms to our list of key words. Thank you for suggesting these terms.

2. *Introduce: the line "First, we use the Web of Science Journal Impact Factor IF", please add the abbreviation and in the rest of the text use only the abbreviation: IF.*

The revised Introduction uses Impact Factor and introduces its abbreviation IF. Subsequently, the manuscript uses IF. Thank you for helping us write concisely.

*After the sentence: We use these scores to compute for each journal-year the two-year and five-year attention score. Third, for each article and each discipline, we compute the correlation coefficient between (1) the two-year impact factor and two-year attention score, and (2) the five-year impact factor and the five-year attention score. The correlation coefficients report three insights.*

*The hypothesis of the study and the purpose of the study need to be added.*

We have added two paragraphs to the revised Introduction. The two paragraphs succinctly state our hypotheses/expectations and the intuition behind them.

3. *Data and methods, in my description you would need to make a drawing - a step by step diagram of the research. A description is good but a drawing always shows the step-by-step nature of the research better.*

The revised manuscript includes Figure 1 on the research process.

*The formula for correlation coefficients (r) should be added.*

The revised manuscript has added the formula for correlation coefficient.

4. *Key Measures
   Table 1 56 Journals, Belonging to 12 Business Disciplines (without the cut in front of belong) or change title: The list of analysed journals in 12 business disciplines*

The revised manuscript has the title that you suggested.

5. *Formula (1 and 2) for Impact Factor . I would use abbreviations and sub-templates to describe them either before the formulae: Impact Factor -IF, time-t, article published in journal - JP (journal paper) etc. Then the formulas would be shorter.*

The revised formulae use abbreviation for Impact Factor to shorten the formulae.

6. *Results: note for Fig. 1 has to be moved under the figure. Under each figure, starting with Figure 2A.....2L you have to write one or two sentences of the conclusion, which is shown in the figure. To each business disciplines because this is the core of the research and requires more analysis.*

The revised manuscript has a note under each figure.

7. *Conclusion, too short, I would add: conclusion of the research and then conclusion. You need to refer to the hypothesis of the research and the purpose of the research, which needs to be added to the introduction first.*

*I wonder if the result of the research should not be a ranking of journals.*

We have revised the Introduction and Conclusion sections.

**Authors’ Responses to Reviewer #2’s Comments**

*A manuscript with a very interesting title. However, the manuscript requires solid refinement, especially from the literature review side.*

The revised manuscript now includes a brief section that reviews the literature. We name the section “The Case for Managerial Relevance of Academic Business Research.”

*The introduction recommends highlighting what is new at work.*

The revised Introduction highlights the novelty.

*The strength is the methodology; the weakness is the very poor review of the literature. The conclusion should also be greatly expanded.*

We have improved the Conclusion section as well. Thank you for helping us improve the manuscript.
